# Bioinspired Architecture Selection for Multitask Learning

**DOI:** 10.3389/fninf.2017.00039

**Published:** 2017-06-22

**Authors:** Andrés Bueno-Crespo, Rosa-María Menchón-Lara, Raquel Martínez-España, José-Luis Sancho-Gómez

**Affiliations:** ^1^Department of Computer Science, Universidad Católica de MurciaMurcia, Spain; ^2^Department of Information and Communications Technologies, Universidad Politécnica de CartagenaCartagena, Spain

**Keywords:** neural networks, multitask learning, architecture design, extreme learning machine, multilayer perceptron

## Abstract

Faced with a new concept to learn, our brain does not work in isolation. It uses all previously learned knowledge. In addition, the brain is able to isolate the knowledge that does not benefit us, and to use what is actually useful. In machine learning, we do not usually benefit from the knowledge of other learned tasks. However, there is a methodology called Multitask Learning (MTL), which is based on the idea that learning a task along with other related tasks produces a transfer of information between them, what can be advantageous for learning the first one. This paper presents a new method to completely design MTL architectures, by including the selection of the most helpful subtasks for the learning of the main task, and the optimal network connections. In this sense, the proposed method realizes a complete design of the MTL schemes. The method is simple and uses the advantages of the Extreme Learning Machine to automatically design a MTL machine, eliminating those factors that hinder, or do not benefit, the learning process of the main task. This architecture is unique and it is obtained without testing/error methodologies that increase the computational complexity. The results obtained over several real problems show the good performances of the designed networks with this method.

## 1. Introduction

The Hebbian learning in neural networks consists in establishing new synapses according to new lived experiences. Thus, this learning is directly related to the so-called structural plasticity which is the brain's ability to alter their physical structure in response to the learning of new information, skills, or habits. This means that when a human being modifies the knowledge about a particular field with new information (both from the same field and from other related fields), new neural connections are established and others are inhibited. This is how human beings can improve knowledge on a specific topic: by incorporating new experiences or related knowledge.

In this context, Multitask Learning (MTL) is a type of machine learning that tries to mimic the structural plasticity of human beings (Baxter, 1993; Caruana, 1995, 1998; Silver and Mercer, 2001). By using a shared representation, the MTL method learns simultaneously a problem (called the main task) along with other related problems (called secondary tasks). Thus, the artificial neural connections obtained by MTL are different from those obtained when the main task is learned by means of a single task learning (STL) scheme. This often leads to a better model for the main task, because there exists a transfer of information from the secondaries to the main task, i.e., the learning of the main task is modified by the information of the secondary tasks. However, in real world applications, it is not always easy to find tasks related with the main one, or to evaluate whether the relationship between them can produce a positive information transfer. Moreover, for machine learning, it is extremely difficult to determine whether the simultaneously training of several tasks can produce a better performance for one of them (considered the main task), in comparison with the result obtained when it is individually trained. This is because a task can contain information that can be helpful or harmful.

In Bueno-Crespo et al. (2015), a method to select related tasks with the main one is presented. Now, this method is used as a part of a new procedure to completely design MTL architectures. A particular pruning connections procedure leads to a positive transfer of information from the secondary tasks to the main task because only the most relevant connections are preserved. In this sense, the proposed method performs a complete design of the MTL networks. To achieve this, the method takes advantage of the benefits of Extreme Learning Machine algorithm (ELM) (Huang et al., 2006), specifically the Optimally Pruned ELM (OP-ELM) (Miche et al., 2010), and the Architecture Selection based on ELM (ASELM) procedures (Bueno-Crespo et al., 2013).

The rest of the paper is organized as follows: Section 2. describes the ASELM algorithm to design Multilayer Perceptrons (MLP). A summarized description of MTL is presented in Section 3. The proposed method is described in Section 4. Section 5 shows the results and finally, conclusions and prospective works close the paper.

## 2. Architecture selection using extreme learning machine

The Extreme Learning Machine (ELM) is based on the concept that if the MLP input weights are fixed to random values, the MLP can be considered as a linear system and the output weights can be easily obtained by using the pseudo-inverse of the hidden neurons outputs matrix **H** for a given training set. Although related ideas were previously analyzed in other works (Pao et al., 1994; Igelnik and Pao, 1997), Huang was the author who formalized it (Huang and Chen, 2007; Huang et al., 2011). He demonstrated that the ELM is an universal approximator for a wide range of random computational nodes, and all the hidden node parameters can randomly be generated according to any continuous probability distribution without any prior knowledge. Thus, given a set of *N* input vectors, a MLP can approximate *N* cases with zero error, $\overset{N}{\underset{i=1}{\sum }}||{\text{y}}_{i}-{\text{t}}_{i}||=0$, being ${\text{y}}_{i}\in {\mathbb{R}}^{m}$ the output network for the input vector ${\text{x}}_{i}\in {\mathbb{R}}^{n}$ with target vector ${\text{t}}_{i}\in {\mathbb{R}}^{m}$. Thus, there exist $\beta_{j}\in {\mathbb{R}}^{m}$, ${\text{w}}_{j}\in {\mathbb{R}}^{n}$ and *b*_*j*_ ∈ ℝ such that,

(1) $$\begin{array}{r} {\text{y}}_{i}=\overset{M}{\underset{j=1}{\sum }}\beta_{j}f\left({\text{w}}_{j}\cdot {\text{x}}_{i}+b_{j}\right)={\text{t}}_{i},\;i=1,\ldots ,N. \end{array}$$

where $\beta_{j}={\left[\beta_{j1},\beta_{j2},\ldots ,\beta_{jm}\right]}^{T}$ is the weight vector connecting the *j*th hidden node with the output nodes, ${\text{w}}_{j}={\left[w_{j1},w_{j2},\ldots ,w_{jn}\right]}^{T}$ is the weight vector connecting the *j*th hidden node with the input nodes, and *b*_*j*_ is the bias of the *j*th hidden node.

For a network with *M* hidden nodes, the previous *N* equations can be expressed by

(2) $$\begin{array}{r} \text{H}\text{B}=\text{T}, \end{array}$$

where

(3) $$\begin{array}{l} \text{H}\left({\text{w}}_{1},\ldots ,{\text{w}}_{M},b_{1},\ldots ,b_{M},{\text{x}}_{1},\ldots ,{\text{x}}_{N}\right)=\\ ={\left[\begin{array}{ccc} f\left({\text{w}}_{1}\cdot {\text{x}}_{1}+b_{1}\right) & \ldots & f\left({\text{w}}_{M}\cdot {\text{x}}_{1}+b_{M}\right)\\ \vdots & \ldots & \vdots \\ f\left({\text{w}}_{1}\cdot {\text{x}}_{N}+b_{1}\right) & \ldots & f\left({\text{w}}_{M}\cdot {\text{x}}_{N}+b_{M}\right) \end{array}\right]}_{N\times M} \end{array}$$

(4) $$\begin{array}{r} \text{B}={\left[\begin{array}{c} \beta_{1}^{T}\\ \vdots \\ \beta_{M}^{T} \end{array}\right]}_{M\times m}\text{and }\text{T}={\left[\begin{array}{c} {\text{t}}_{1}^{T}\\ \vdots \\ {\text{t}}_{N}^{T} \end{array}\right]}_{N\times m} \end{array}$$

where **H** ∈ ℝ^*N*×*M*^ is the hidden layer output matrix of the MLP, **B** ∈ ℝ^*M*×*m*^ is the output weight matrix, and **T** ∈ ℝ^*N*×*m*^ is the target matrix of the *N* training cases. Thus, as **w**_*j*_ and *b*_*j*_ with *j* = 1, …, *N*, are randomly selected, the MLP training is given by the solution of the least square problem of Equation (2), i.e., the optimal output weight layer is $\hat{\text{B}}={\text{H}}^{‡}\text{T}$, where **H**^‡^ is the Moore-Penrose pseudo-inverse (Serre, 2002).

ELM for training MLPs can be therefore summarized as shown in Algorithm 1.

**Table:** **Algorithm 1** Extreme Learning Machine (ELM)

	Given a training set $D=\{(x_{i},t_{i})|x_{i}\in {\mathbb{R}}^{n},t_{i}\in {\mathbb{R}}^{m},i=1,\ldots ,N\}$, an activation function *f* and an hidden neuron number *M*,
1:	Assign arbitrary input weights **w**_*j*_ and biases *b*_*j*_, *j* = 1, …, *M*.
2:	Compute the hidden layer output matrix **H** using Equation (3).
3:	Calculate the output weight matrix **B** = **H**^‡^**T**, where **B** and **T** are both defined in Equation (4).

ELM provides a fast and efficient MLP training (Huang et al., 2006), but it needs to fix the number of hidden neurons to obtain a good generalization capability. In order to avoid the exhaustive search for the optimal value of *M*, several pruned methods have been proposed (Mateo and Lendasse, 2008; Miche et al., 2008a,b; Rong et al., 2008; Miche and Lendasse, 2009; Miche et al., 2010). Among them, the most commonly used is the ELM Optimally Pruned (OP-ELM) (Miche et al., 2010). The OP-ELM sets a very high initial number of hidden neurons (*M*≫*N*) and, by using Least Angle Regression algorithm (LARS) (Similä and Tikka, 2005), sorts the neurons according to their importance to solve the problem (Equation 2). The pruning of neurons is done by utilizing Leave-One-Out Cross-Validation (LOO-CV) and choosing the combination of neurons (which have been previously sorted by the LARS algorithm) that provides lower LOO error. The LOO-CV error is efficiently computed using the Allen's formula (Miche et al., 2010). For more detail, a summary of the OP-ELM algorithm is shown in Algorithm 2 (García-Laencina et al., 2011).

**Table:** **Algorithm 2** Optimally Pruned-ELM (OP-ELM)

	Given a training set $D=\{(x_{j},t_{j})|x_{j}\in {\mathbb{R}}^{n},t_{j}\in {\mathbb{R}}^{m},j=1,\ldots ,N\}$, a mix of activation functions (sigmoid, gaussian, and linear), and a large number of neurons *M*,
1:	Randomly assign input weights ${\left\{{\text{w}}_{i},b_{i}\right\}}_{i=1}^{M}$.
2:	Calculate the hidden layer output matrix **H** using **X** and input weights.
3:	Ranking the hidden outputs using the MRSR algorithm, i.e., **H** is ranked, and set **H**^0^ as an empty matrix.
4:	**for** *k* = 1 to *N* **do**
5:	Add the *k*-th node to the model → ${\text{H}}^{k}=\left[{\text{H}}^{k-1},{\text{h}}_{k}\right]$, being **h**_*k*_ the *k*-th column of **H**.
6:	Computes LOO error ($\epsilon_{k}^{PRESS}$) with **H**^*k*^.
7:	**end for**
8:	Select the network size (M^*^) according to $\epsilon_{PRESS}^{M^{*}}<\epsilon_{PRESS}^{k},\forall k\in \left(1,2,\ldots ,M\right)$.
9:	Calculate the output weights matrix: B^*^ = (H^*^)^‡^**T**.

Recently, a new method to design MLP architectures has been presented in Bueno-Crespo et al. (2013). It is called ASELM (“Architecture Selection Using Extreme Learning Machine”) and is based on the OP-ELM. Thus, once the initial MLP architecture is defined, the OP-ELM optimally discards those hidden neurons whose combination of input variables is not relevant to the target task. Because of the binary value of the input weights, the selection of hidden nodes implies also the selection of those relevant connections between the input and hidden layers. Thus, only input connections corresponding to selected hidden neurons and with input weights values equal to 1 will be part of the final architecture. A summary of the ASELM algorithm is shown below (Algorithm 3).

**Table:** **Algorithm 3** Architecture Selection ELM (ASELM)

	Given a training set $D=\{(x_{i},t_{i})|x_{i}\in {\mathbb{R}}^{n},t_{i}\in {\mathbb{R}}^{m},i=1,\ldots ,N\}$, activation function *f*, an hidden neuron number 2^*n*^−1, where *n* is the number of input features, proceed as follows:
1:	The weights of the input layer are initialized with binary values by considering all possible combinations of inputs. The case of all weights set to zero is discarded.
2:	MLP network is trained by the OP-ELM and, then, useless hidden neurons are discarded according to the ranking given by LARS and LOO-CV procedure.
3:	The final MLP architecture is given by the selected hidden neurons with its corresponding input(s) weight(s) equal to one.

## 3. Multitask learning architecture

**Figure 1:**
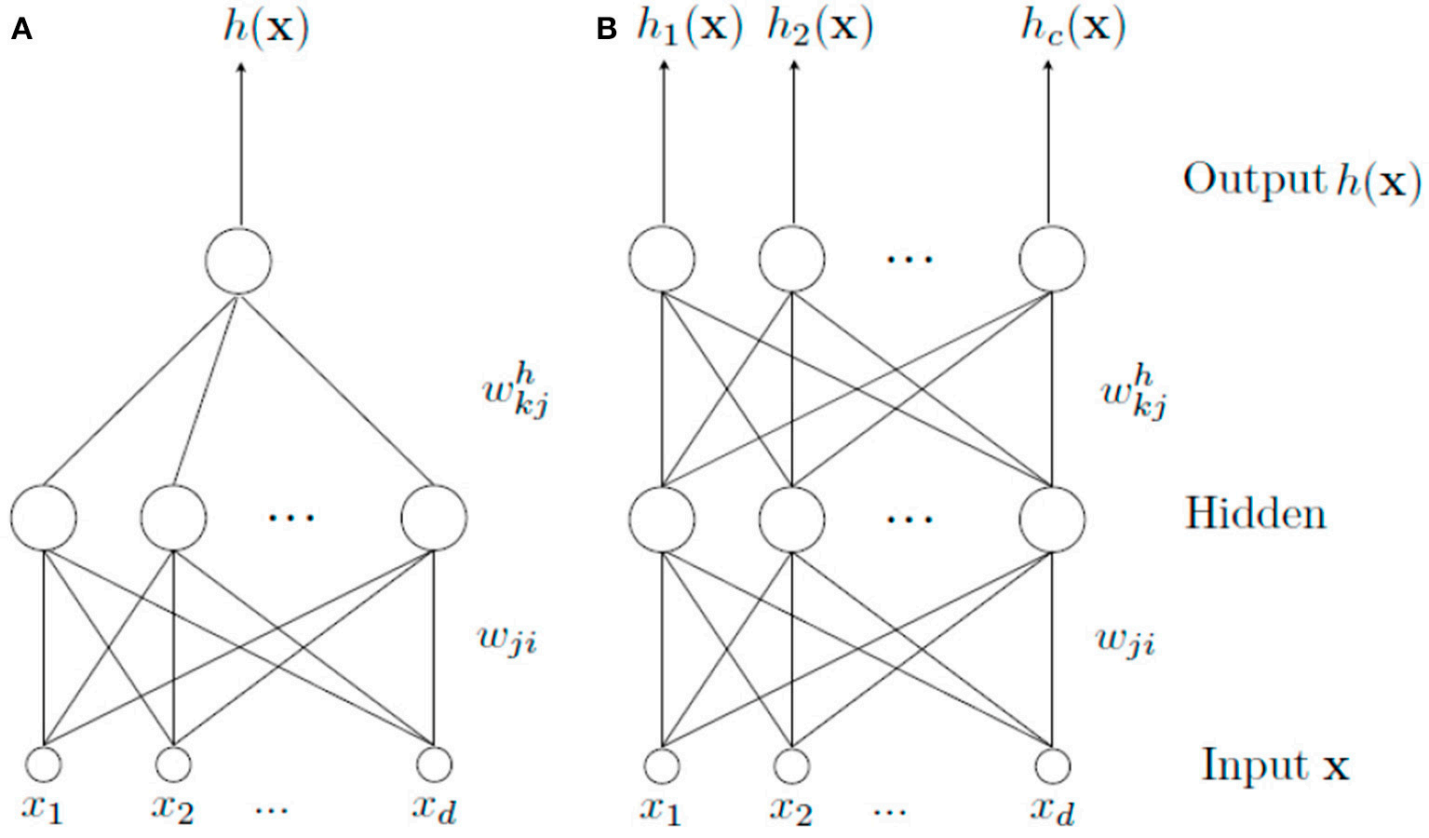
Different learning schemes. In **(A)**, a STL architecture is shown. It is used to solve a single task alone. In **(B)**, a set of tasks are learned simultaneously by means of a MTL architecture. In this case, there is a common part (from the input to the hidden layer) and a specific part (from the hidden layer to the output) for each task.

The MTL architecture for a neural network is similar to the classical scheme STL (Single Task Learning). They differ in that MTL scheme has an output for each task to be learned, whereas STL scheme has a separate network for each one (Figure 1). Thus, when we speak about MTL, we are referring to a type of learning where a main task and other tasks (considered as secondary tasks) are learned all at once in order to help learning of the main one.

In a MTL scheme, there is a common part shared by all tasks and a specific one for each task. The common part is formed by the weights connections from the input features to the hidden layer, allowing common internal representation for all tasks (Caruana, 1993). Thanks to this internal representation, learning can be transferred from one task to another (Caruana, 1998). The specific part, formed by the weights that connect the hidden layer to the output layer, specifically allows modeling each task from the common representation. The main problem with this type of learning is to find tasks related to the main one. Even in case of finding them, it may be difficult to know the kind of relationship they have, because it can be a positive or negative influence to learn the main task.

## 4. Proposed method

The method proposed in this paper is called *MTL*_*ASELM*_ since it is based on the ASELM to design MTL architectures. To do this, it is necessary to introduce a couple of modifications to the original method so as to adapt it to MTL. Firstly, the targets of secondary tasks will be used as new input features (removing them from the outputs of the classic MTL scheme) so that a similar architecture to that shown in Figure 1A is obtained. There is only a single output corresponding to the main task and an input vector composed now by the original input features and the targets of secondary tasks. This network is designed and trained using ASELM which, as it was commented before, realizes a selection of hidden nodes that implies also the selection of those relevant connections between the input and hidden layer. The selection of relevant secondary tasks is now performed since they are part of the input vector.

**Figure 2:**
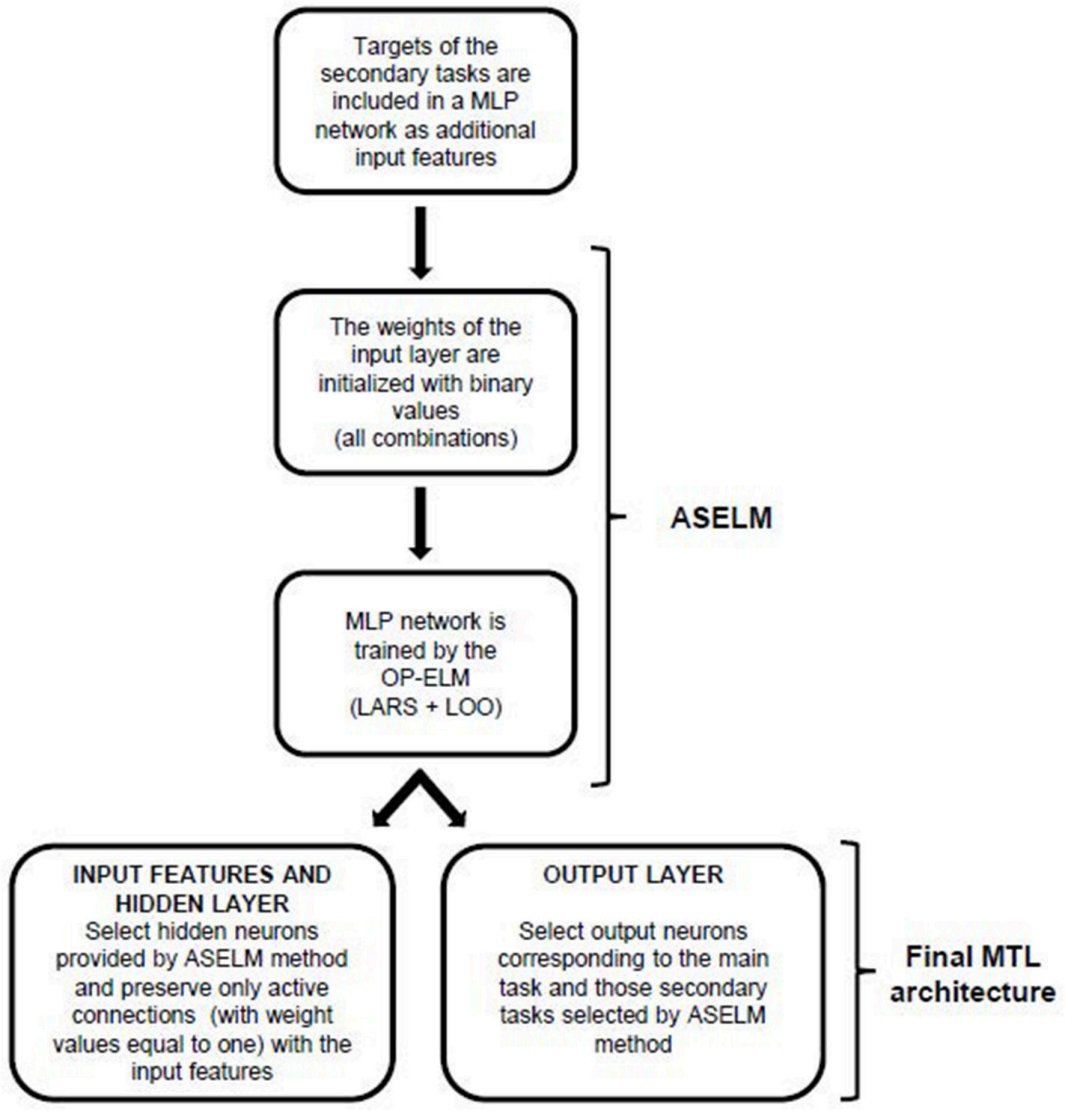
Flowchart of the proposed method.

In a second stage, a MTL architecture is created. The secondary tasks selected in the previous stage as the most relevant to learn the main task are included as output components in the MTL neural network. A scheme of the proposed method can be seen in Figure 2.

This idea of exchanging outputs for inputs is not new. Caruana proposed that some inputs may work better as outputs, i.e., as new secondary tasks (Caruana, 1998). This idea is very interesting in machine learning and it has been used, for example, for developing efficient procedures to classify patterns with missing data (García-Laencina et al., 2010, 2013).

*MTL*_*ASELM*_ method allows pruning to take place both at the hidden layer and the output layer, at the same time that provides a unique solution. This uniqueness comes from the binary initialization of the hidden weights, which eliminates the random component thereof.

To further clarify the *MTL*_*ASELM*_ method, the following section includes an example of how the method is applied step by step to solve a particular problem (Logic Domain problem).

## 5. Experiments

In order to show the goodness of the *MTL*_*ASELM*_ method for designing an MTL architecture, results of classification test obtained with the single-task learning (STL), classic multitask learning (MTL), and *MTL*_*ASELM*_ have been compared. While the *MTL*_*ASELM*_ architecture is directly obtained by the proposed method, the best architecture for STL and MTL has been selected by cross-validation. For experiments, the three architectures are trained using the stochastic back-propagation with a cross-validation with 10-fold × 30 initializations. “Logic Domain,” “Monk's Problems,” “Telugu,” “Iris Data,” and “User Knowledge Modeling” datasets, will be used to show the performance of the method. These data sets are available at the UCI ML Repository (Asuncion and Newman, 2007), excepting “Logic Domain” problem (McCracken, 2003). Specific details about results for each dataset are described below.

“Logic Domain” dataset is used to see how MTL architecture is created by the *MTL*_*ASELM*_ method. This dataset is a toy problem specially designed for multitask learning. In this problem, targets are represented by the combination of four real variables (from seven inputs: *x*_1_,…,*x*_7_), considering the first task as the main task, and the others as secondary ones.

Table 1 shows the logical expression for each task. Note, that the main task (*T*_*p*_) is only determined by the first four features of the problem. The secondary tasks share one or more variables with the main one. Nevertheless, only the second secondary task (*T*_*Sec*_2__) shares a common logic subexpression (*x*_3_ > 0.5 ∨ *x*_4_ > 0.5) with the main task.

**Table 1:** Description of the “Logic Domain” tasks.

**Task**	**Logical expression**
*T*_*P*_	(*x*_1_ > 0.5 ∨*x*_2_ > 0.5) ∧ (*x*_3_ > 0.5 ∨*x*_4_ > 0.5)
*T*_*Sec*_1__	(*x*_2_ > 0.5 ∨*x*_3_ > 0.5) ∧ (*x*_4_ > 0.5 ∨*x*_5_ > 0.5)
*T*_*Sec*_2__	(*x*_3_ > 0.5 ∨*x*_4_ > 0.5) ∧ (*x*_5_ > 0.5 ∨*x*_6_ > 0.5)
*T*_*Sec*_3__	(*x*_4_ > 0.5 ∨*x*_5_ > 0.5) ∧ (*x*_6_ > 0.5 ∨*x*_7_ > 0.5)

*Each task is described by a logical combination of four input features*.

**Figure 3:**
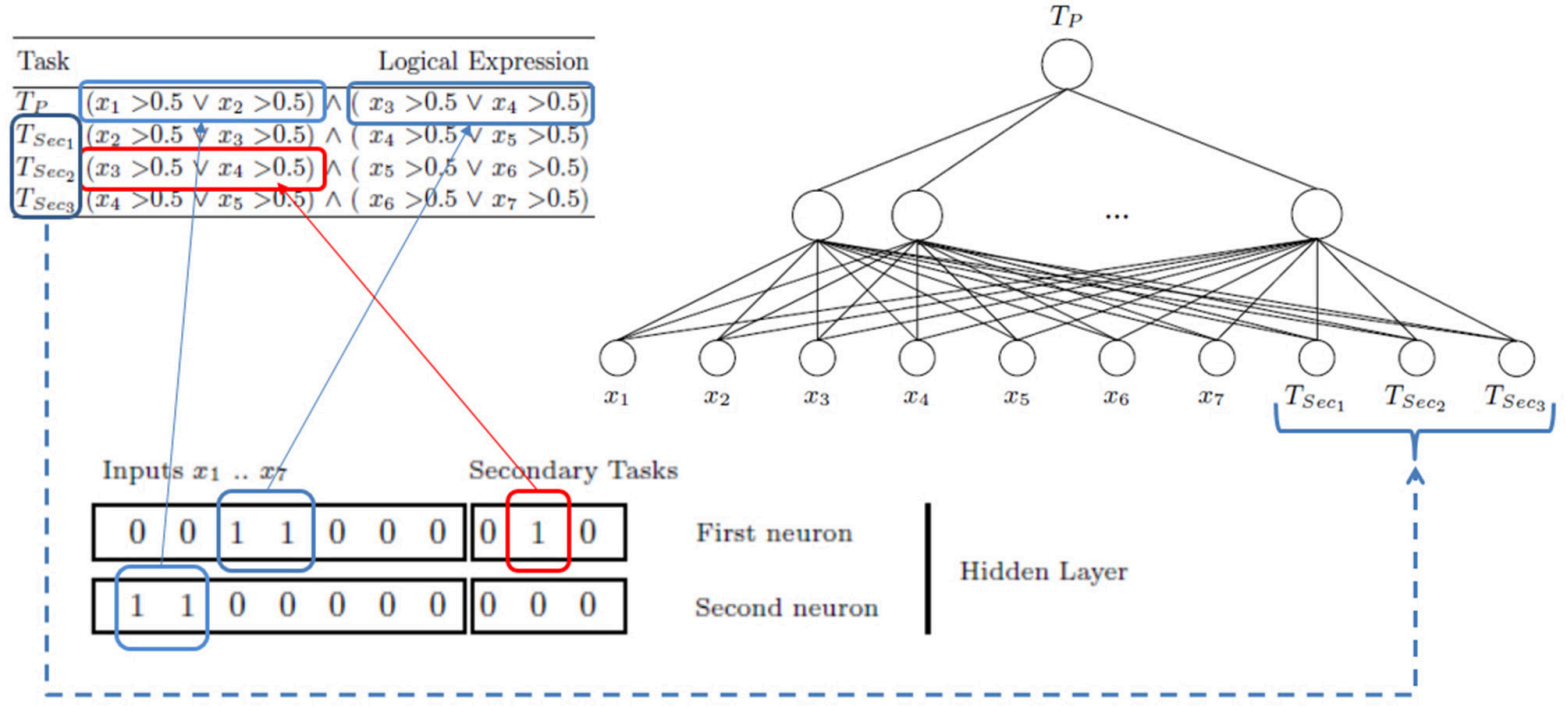
Logic Domain problem. Scheme to learn the main task using secondary tasks as inputs. 1023 neurons in the hidden layer have been generated. After ASELM is applied only two hidden neurons are selected whose weight vectors are shown in black boxes. The first neuron has three connections corresponding to the input features *x*_3_ and *x*_4_ and the second secondary task (*T*_*Sec*_2__). The second neuron is represented only by the input features *x*_1_ and *x*_2_.

Initially, the neural network architecture is composed by *M* = 1023 (2^*n*^ − 1, with *n* = 10; seven input features + three extra features corresponding to three secondary tasks) hidden units (see Figure 3). This suppose a large enough hidden layer number according to the ELM theory. Once this model is trained with ASELM method, the result is quite significant. The ASELM selects only two hidden neurons as the most relevant to learn the main task (Bueno-Crespo et al., 2013). By relevance order, these hidden weights are **w**_194_ = [0 0 1 1 0 0 0 0 1 0] and **w**_768_ = [1 1 0 0 0 0 0 0 0 0] corresponding to hidden neuron number 194 and 768, respectively. For simplicity, we will be referred to them as first neuron or **w**_1_ and second neuron or **w**_2_. From **w**_1_, it can be observed that the first selected hidden neuron is only connected to input features *x*_3_ and *x*_4_, as well as the second secondary task (*T*_*Sec*_2__). From **w**_2_, it follows that only *x*_1_ and *x*_2_ contribute to learning through their connection to the second hidden neuron (see Figure 3).

**Figure 4:**
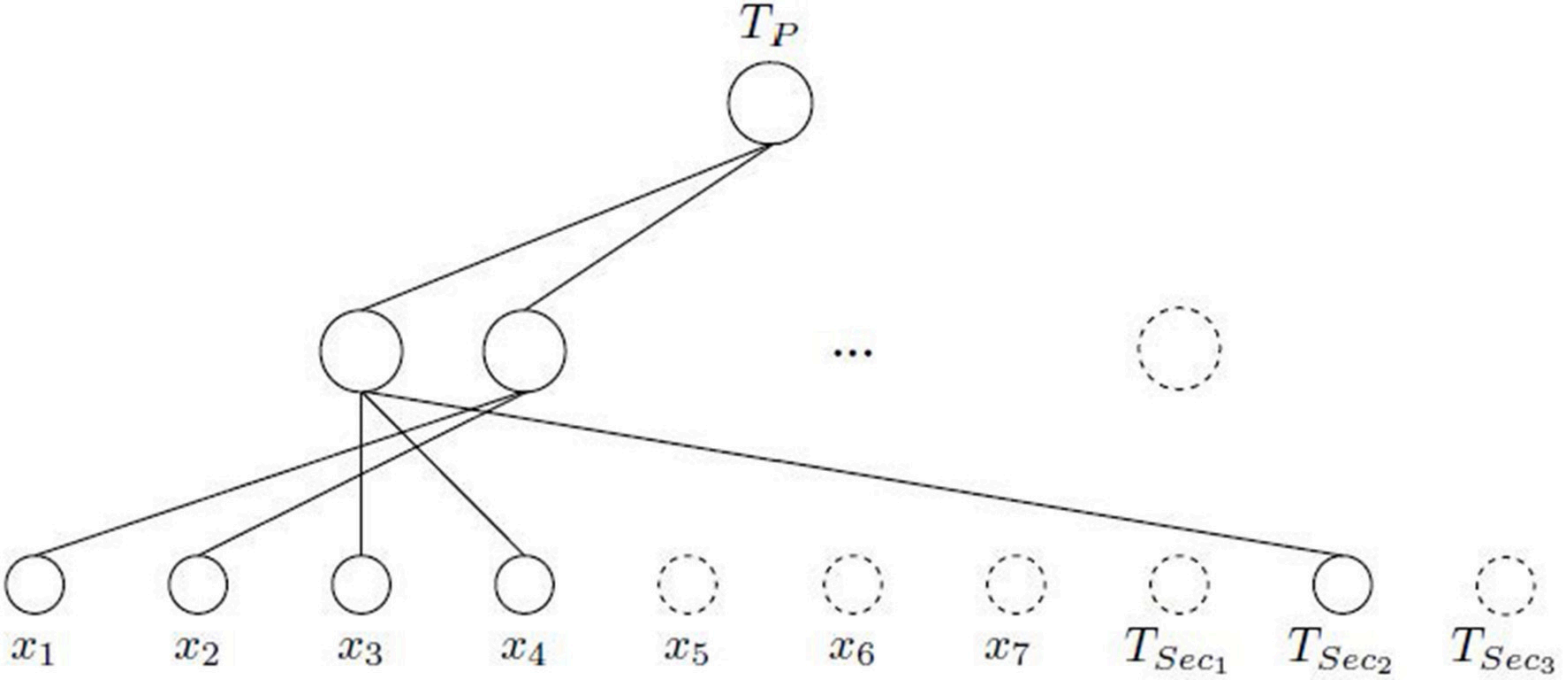
Logic Domain problem. Intermediate scheme where connections are pruned after ASELM. It can be observed how the input features *x*_5_, *x*_6_, and *x*_7_, all hidden nodes least two, and secondary tasks *T*_*Sec*_1__ and *T*_*Sec*_3__ are removed because they are irrelevant for the learning of the *T*_*P*_.

This means that only *T*_*Sec*_2__ is influencing in the learning of the *T*_*P*_ through the first neuron that learns the input features *x*_3_ and *x*_4_, which is an expected result according to the previous comment indicating the relationship between *T*_*p*_ and *T*_*sec*_2__ (see Table 1). The second selected hidden neuron is only composed by the input features *x*_1_ and *x*_2_, without any input connection from the secondary tasks. Figure 4 shows the final architecture given by ASELM method.

**Figure 5:**
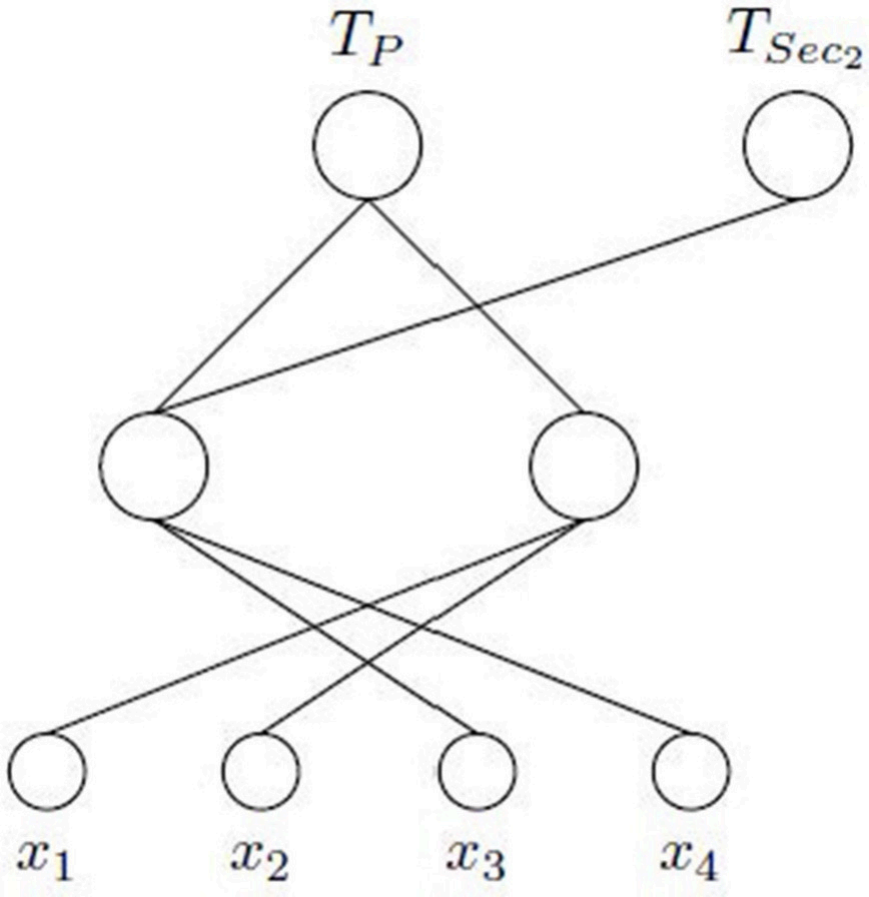
Logic Domain problem. Final architecture obtained with *MTL*_*ASELM*_. *T*_*P*_ shares a hidden layer neuron with *T*_*Sec*_2__, which learns the common part of both tasks.

Next, a MTL architecture is created considering as outputs those corresponding to the main task and secondary ones selected in the previous stage. The latter are incorporated into the output layer preserving the connections established by the ASELM. In our case, only *T*_*Sec*_2__ has been selected. Figure 5 shows the final *MTL*_*ASELM*_ architecture. *MTL*_*ASELM*_ has removed the input features *x*_5_, *x*_6_, and *x*_7_, and has selected only 2 neurons in the hidden layer from the 1023 neurons initially considered.

**Figure 6:**
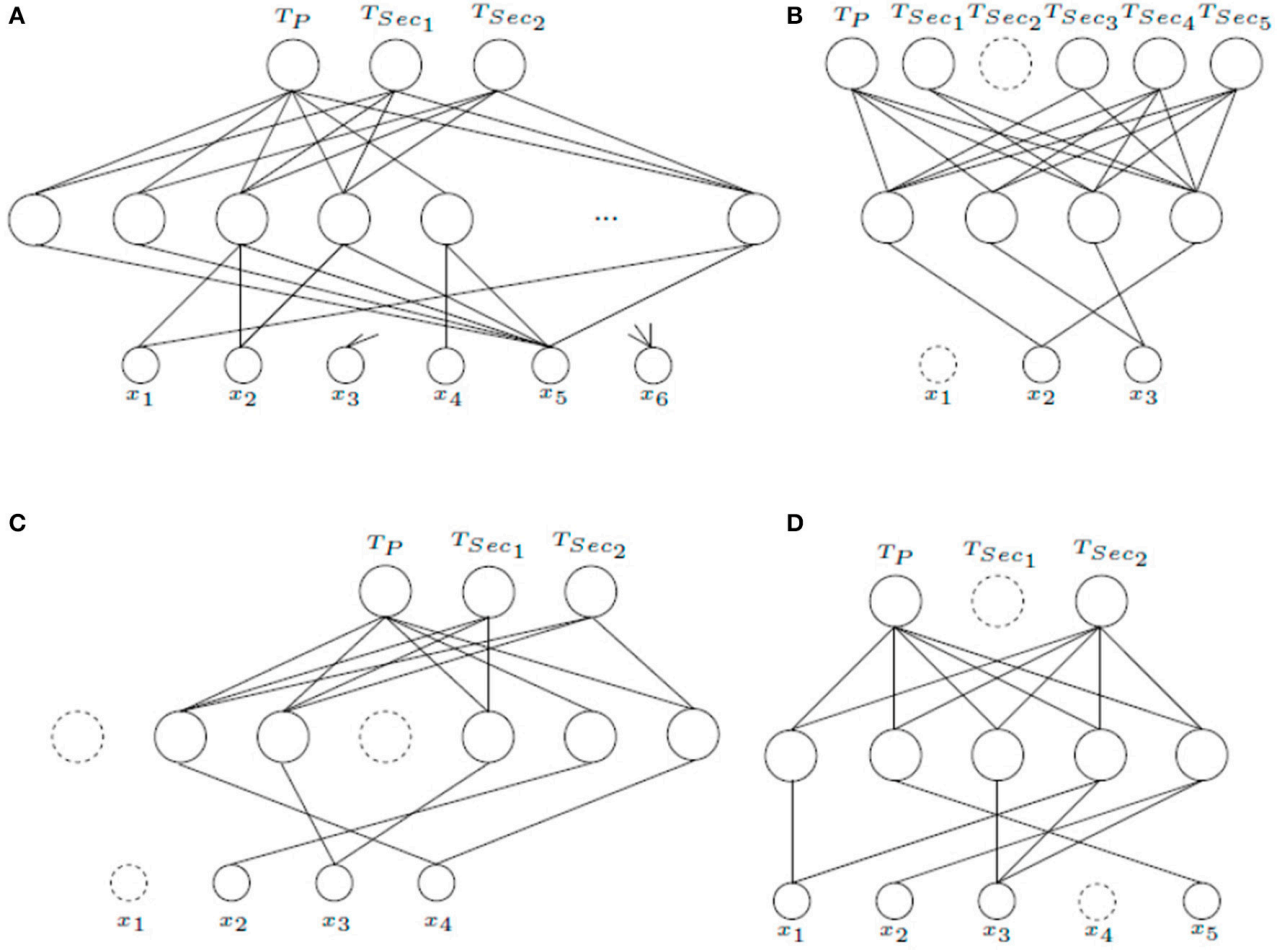
Final *MTL*_*ASELM*_ architectures for “Monk's Problems” **(A)**, “Telugu” **(B)**, “Iris Data” **(C)**, and “User Knowledge Modeling” **(D)**.

Figure 6 shows the *MTL*_*ASELM*_ schemes for other studied datasets. “Monk's Problems” dataset is a collection of three toy problems that present the same domain (six input features). In this problem, the targets associated to each task are described by the logical relations. Thus, Monk 1 (*T*_*P*_) is described by (*x*_1_ = *x*_2_) ∨ (*x*_5_ = 1); in Monk 2 (*T*_*Sec*_1__) exactly two identities from *x*_1_ = 1, *x*_2_ = 1, *x*_3_ = 1, *x*_4_ = 1, *x*_5_ = 1, *x*_6_ = 1 must be satisfied; and in Monk 3 (*T*_*Sec*_2__), (*x*_5_ = 3 and *x*_4_ = 1) or (*x*_5_ ≠ 4 and *x*_2_ ≠ 3) have to be fulfilled. *MTL*_*ASELM*_ selects 14 neurons in the hidden layer from a total of 255. Figure 6A presents the first five neurons and the last one for the selected architecture. For example, if we observe the first neuron, it connects the input feature *x*_5_ with the outputs of *T*_*P*_ and *T*_*Sec*_1__, but not with *T*_*Sec*_2__. It can be observed that target associated to *T*_*P*_ and *T*_*Sec*_1__ match the value of *x*_5_, what does not happen for *T*_*Sec*_2__.

“Telugu” language dataset represents one of six languages designated a classical language of India. This datasets consists of three input features that represent language formants. For “Telugu,” *MTL*_*ASELM*_ selects 4 neurons in the hidden layer from a total of 255 initial neurons. Figure 6B shows the final architecture obtained. As can be seen, this architecture uses only two of the three input features, what is quite interesting because in dialects with fewer than six vowels, two formants are only required to classify (Pal and Majumder, 1977).

“Iris Data” (Figure 6C) represent a dataset of three types of flowers represented by four input features. For this dataset, 5 neurons are selected in the hidden layer from a total of 63 neurons. It can be observed that the input feature *x*_1_ has been removed. The results show that the proposed method has a much more simplified architecture than classical multitask learning, although the classification test is similar due to the simplicity of the problem (see Table 2).

**Table 2:** Classification Test “CT” (mean ± standard deviation) with different schemes on several datasets.

**Dataset**	**Scheme**	**CT (mean ±std)**	**Removed**
Logic Domain	*STL*	0.730 ± 0.038	
	*MTL*	0.702 ± 0.026	
	*MTL*_*ASELM*_	0.746 ± 0.035	*x*_5_, *x*_6_, and *x*_7_
Monk's Problems	*STL*	0.954 ± 0.016	
	*MTL*	0.982 ± 0.026	
	*MTL*_*ASELM*_	0.991 ± 0.016	none
Telugu	*STL*	0.836 ± 0.012	
	*MTL*	0.841 ± 0.011	
	*MTL*_*ASELM*_	0.866 ± 0.026	*x*_1_
Iris Data	*STL*	0.978 ± 0.005	
	*MTL*	0.973 ± 0.014	
	*MTL*_*ASELM*_	0.970 ± 0.003	*x*_1_
User Knowledge Modeling	*STL*	0.913 ± 0.019	
	*MTL*	0.928 ± 0.036	
	*MTL*_*ASELM*_	0.950 ± 0.006	*x*_4_

*For MTL_ASELM_, removed inputs are shown*.

“User Knowledge Modeling” (Figure 6D) is the real dataset about the students' knowledge status about the subject of Electrical DC Machines. The target is represented by four levels (very low, low, middle, and high). To give a multitasking approach a pairwise combination has been made (*T*_*P*_ = (very low ∨ low), *T*_*Sec*_1__ = (low ∨ middle), and *T*_*Sec*_2__ = (middle ∨ high)). It can be observed that *T*_*Sec*_1__ is removed. It is because *T*_*Sec*_2__ is more important to *T*_*P*_, since *T*_*Sec*_2__ represents its opposite. Finally, 5 neurons are selected in the hidden layer from a total of 127 initial neurons.

Table 2 shows the classification accuracy results for all the data sets. Because the Logic Domain is an easy problem to solve for an MLP in an STL scheme, the number of samples has been reduced to 50 so that multitask learning can be appreciated. With all training samples, the result between STL and *MTL*_*ASELM*_ is practically invaluable. Taking into account this reduction of samples for the Logic Domain problem, *MTL*_*ASELM*_ presents better classification accuracy than STL and MTL. However, STL is better than the classic MTL, since MTL presents a completely interconnected scheme that is positively influenced by the related task (*T*_*Sec*_2__) and negatively by unrelated tasks (*T*_*Sec*_1__ y *T*_*Sec*_3__). This is not a general rule but it is an empirical result that shows that there are tasks that help the main task and others that are harmful.

For the rest of the data sets, the *MTL*_*ASELM*_ always provides the best results on average with a low standard deviation. This robustness in the solution is due to the particular initialization of the hidden weights that *MTL*_*ASELM*_ realizes.

To validate this assertion, a non-parametric statistical test has been performed. Specifically, the Wilcoxon Signed Ranks Test is used (Kruskal, 1957). A peer review has been performed. Comparing *MTL*_*ASELM*_ to STL, the *p*-value obtained is 0.078, which indicates that there are significant differences to 92%, being the best *MTL*_*ASELM*_. Likewise, when applying the test with *MTL*_*ASELM*_ against classic MTL, the *p* = 0.080 indicates that there are significant differences to 92%, being *MTL*_*ASELM*_ better than MTL. However, there are no significant differences between STL and MTL, since the *p* = 0.683.

## 6. Discussion and future work

This paper presents a method to select tasks to be used in a MTL scheme providing information about weight connection, hidden nodes, input features, and most helpful secondary tasks for the learning of the main task. This method has been named *MTL*_*ASELM*_ because it is based on the ASELM algorithm (Bueno-Crespo et al., 2013), which proves to be an efficient method and single solution for the complete design of a MLP (input features, weights connections, and hidden nodes). By using secondary tasks as input features, *MTL*_*ASELM*_ applies the ASELM on the initial network which only has a single output corresponding to the main task. Thus, irrelevant nodes and connections are eliminated, what implies a selection of features (among which are the secondary tasks). After this stage, a final network is built with a dimension in the output layer equal to the number of secondary tasks selected as relevant plus one. Thus, the main drawback of multitask learning is eliminated, i.e., the negative influence of unrelated tasks. In addition, the modification of ASELM method to adapt it for a multitask scheme is achieved not only to eliminate connections from inputs features to hidden layer, but also from hidden to output layer. It is worth highlighting that, as well as ASELM, the *MTL*_*ASELM*_ method provides a single solution. This is due the fact that a binary initial selection of the hidden weights substitutes any random initialization process. Another important advantage is that it requires no parameter to be configured by the user. In the experiments section, it has been observed over real problems that the method *MTL*_*ASELM*_ gets a simplified solution with good generalization capabilities, in comparison to those obtained by a fully connected solution given by the classic MTL scheme.

Authors are working on extending the method to other learning models, such as Radial Basis Functions (RBF). Applying *MTL*_*ASELM*_ to regression problems is another research field since the ASELM is optimized for classification according to Huang et al. (2010). This limitation of the present method is due to the nature of ELM method, which is based on the pseudoinverse calculation. In this regard, we are working to use the sequential calculation pseudoinverse of Moore-Penrose (Van Heeswijk et al., 2011; Tapson and Van Schaik, 2013). Another line of research is to extend this method in the field of Deep Learning since new works on MultiTask Learning have lately appeared, most of them within the scope of Deep Learning (Liu et al., 2015; Thanda and Venkatesan, 2017).

## Author contributions

AB: Proposed method, software programming, experiments, reviews, results, and conclusions. RMML: Introduction, software programming, contributions of ideas and reviews. RME: Method description, contributions of ideas and reviews. JS: Work direction, architecture selection, contributions of ideas, reviews, conclusions, and future work.

### Conflict of interest statement

The authors declare that the research was conducted in the absence of any commercial or financial relationships that could be construed as a potential conflict of interest. The handling Editor declared a shared affiliation, though no other collaboration, with several of the authors RMML and JS, and the handling Editor states that the process met the standards of a fair and objective review.
